# Characterization of the Interaction between Eupatorin and Bovine Serum Albumin by Spectroscopic and Molecular Modeling Methods

**DOI:** 10.3390/ijms140714185

**Published:** 2013-07-09

**Authors:** Hongliang Xu, Nannan Yao, Haoran Xu, Tianshi Wang, Guiying Li, Zhengqiang Li

**Affiliations:** 1 Key Laboratory for Molecular Enzymology and Engineering of the Ministry of Education, College of Life Sciences, Jilin University, Changchun 130012, China; E-Mails: xuhlxu@hotmail.com (H.X.); yaonnyao@outlook.com (N.Y.); xuhr10@mails.jlu.edu.cn (H.X.); tiamshi@126.com (T.W.); ligy@jlu.edu.cn (G.L.); 2 School of Life Science and Technology, Mudanjiang Normal University, Mudanjiang 157011, China

**Keywords:** eupatorin, bovine serum albumin, binding, spectroscopy, molecular modeling

## Abstract

This study investigated the interaction between eupatorin and bovine serum albumin (BSA) using ultraviolet-visible (UV-vis) absorption, fluorescence, synchronous fluorescence, circular dichroism (CD) spectroscopies, and molecular modeling at pH 7.4. Results of UV-vis and fluorescence spectroscopies illustrated that BSA fluorescence was quenched by eupatorin via a static quenching mechanism. Thermodynamic parameters revealed that hydrophobic and electrostatic interactions played major roles in the interaction. Moreover, the efficiency of energy transfer, and the distance between BSA and acceptor eupatorin, were calculated. The effects of eupatorin on the BSA conformation were analyzed using UV-vis, CD, and synchronous fluorescence. Finally, the binding of eupatorin to BSA was modeled using the molecular docking method.

## 1. Introduction

**Scheme 1:**
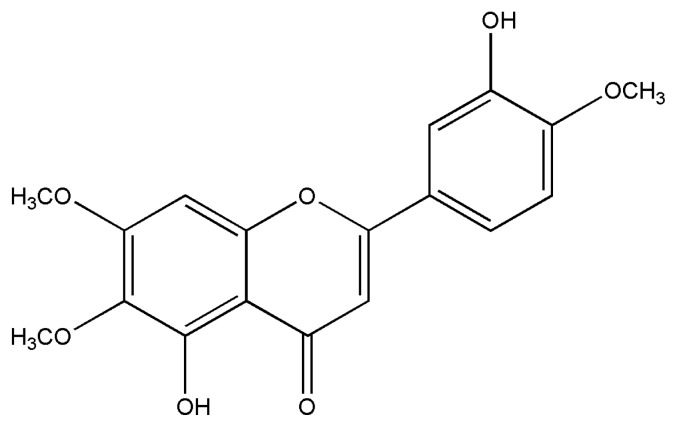
The chemical structure of eupatorin.

Flavonoids constitute a class of natural compounds that possess a myriad of physiological functions, including antiproliferative activity in tumor cell lines, antioxidant, and anti-inflammatory activities. Extensive investigations of flavonoids over the past few decades have identified the various effects of flavonoids on human cell physiology and gene expression [1,2]. As a group, flavonoids have aroused extensive interest in fields investigating tumor prevention and treatment, and among flavonoids, eupatorin (Scheme 1) has received the greatest attention worldwide. Many studies have demonstrated that eupatorin possesses obvious and strong inhibitory effects on proliferation of MCF-7, MDA-MB-468, MK-1, HeLa, B16F10, and A431 cells, *etc*. [1–4]. It has even been shown that submicromolar concentrations of eupatorin could strongly inhibit MDA-MB-468 cell proliferation. Results of *in vitro* assays showed that eupatorin could act as a potential chemopreventive agent and provide desired selectivity between cancer and normal cells, which potentially offers a solution to an obstacle within standard cytotoxic chemotherapeutic drugs [2]. Lately, it has been reported that eupatorin could inactivate the mitotic checkpoint, leading to polyploidy and apoptosis, which might constitute one of the mechanisms responsible for its antiproliferative effect in tumor cells [5].

It is well established that the drug-protein interaction extensively influences the biological activity of a drug. Bovine serum albumin (BSA), an extensively studied protein, is one of the most important carriers for a broad spectrum of exogenous and endogenous ligands, and therefore is widely used as a model protein in investigations of drug-protein interactions [6,7]. BSA is a 66.4 KDa globular heart-shaped protein composed of 583 amino acid residues. It contains three homologous domains (I, II, and III), which are divided by 17 disulfide bonds into nine loops (L1–L9). The loops in each domain consist of a sequence of large-small-large loops that form a triplet. Each domain, in turn, is composed of two subdomains (IA, IB, *etc*.). X-ray crystallographic data have indicated that the albumin structure is predominantly α-helix, and the remaining polypeptide occurs in turns and extended or flexible regions between subdomains with no β-sheet [8–11]. The BSA binding sites for endogenous and exogenous ligands may be located in subdomains IIA and IIIA, known as Sudlow’s sites I and II, respectively, and drug binding sites are often located in these domains [12]. BSA has two tryptophan residues (Trp134 and Trp213) that possess intrinsic fluorescence [11,13]. Trp134 is situated on the surface of the protein, and Trp213 is located within a hydrophobic binding pocket of the molecule [14].

Among the several physiological functions of eupatorin, its excellent antiproliferative effect on tumor cells has attracted considerable interest. However, extensive eupatorin investigations have tended to focus on its isolation, identification, and cytological functions. Meanwhile, few reports have studied the interaction between eupatorin and proteins. In the present study, we characterized the interaction between eupatorin and BSA *in vitro* under physiological conditions, via ultraviolet-visible (UV–vis), fluorescence, CD, and molecular modeling methods. Binding parameters, such as the binding constant, number of binding sites, binding force, and binding distance, were obtained from the fluorescence data. In order to obtain the most accurate data, we apply a correction for the inner effect, which may affect the binding parameters calculated from the fluorescence data. Further, we monitored the effects of eupatorin on BSA secondary structure changes using the approaches described above. In addition, molecular modeling was employed to estimate the participation of specific chemical groups, as well as their interactions in complex stabilization, at a molecular level. The accurate and comprehensive data accumulated here has helped to elucidate the binding mechanism between eupatorin and BSA. It has also contributed to an enhanced understanding of the transportation, distribution, metabolism, and drug efficacy of eupatorin in blood, and also played a valuable insight in the renovation, design, efficacy, and security enhancement of eupatorin.

## 2. Results and Discussion

### 2.1. UV–vis Absorption Spectra Experiments

**Figure 1:**
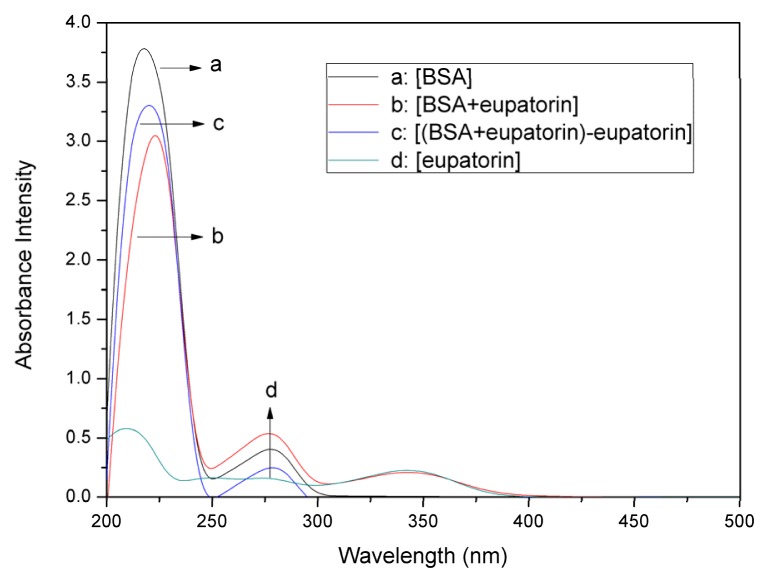
Ultraviolet-visible (UV–vis) spectra of BSA in the presence of eupatorin (*T* = 298 K, pH 7.4): (**a**) Absorption spectrum of bovine serum albumin (BSA) only; (**b**) Absorbance spectrum of eupatorin—BSA when the molar ratio is 1:1; (**c**) Difference absorption spectrum between eupatorin—BSA and eupatorin; (**d**) Absorption spectrum of eupatorin only. *C*_BSA_ = *C*_eupatorin_ = 1.0 × 10^−5^ M.

UV–vis absorption measurement is a simple and pertinent method that is used to investigate structural changes and to explore complex formation [15,16]. It is well accepted that dynamic quenching affects only the excited states of the fluorophores, while has a scarce effect on the absorption spectrum [17]. To initially verify the quenching mechanism, the UV–vis absorption spectra of (BSA), (eupatorin), (BSA + eupatorin), and ((BSA + eupatorin)-eupatorin) were measured and recorded. As shown in Figure 1, BSA possessed two absorption peaks at 220 nm and 280 nm. The strong absorption peak at around 220 nm reflected the absorption of the backbone of BSA, while the weak absorption peak at around 280 nm resulted from the aromatic amino acids (Trp, Tyr, and Phe). These findings were in accordance with those of other studies [18–21]. Eupatorin exhibited two evident peaks at 220 nm and 340 nm. With the addition of eupatorin, the intensity of the peak at 220 nm was dramatically changed with a red shift of about 2 nm, indicating disturbances to the microenvironment around the amide bonds in the protein. The results demonstrated the existence of an interaction between eupatorin and BSA. The absorption spectrum at around 280 nm did not shift, indicating that the microenvironment around the aromatic acid residues was not exposed to any change upon BSA-eupatorin complexation. Collectively, the fluorescence quenching of BSA by drug was caused by the static quenching, which supported the BSA UV spectral shifts [17].

### 2.2. Fluorescence Quenching Studies of BSA

Thus far, fluorescence spectroscopy has been regarded as the most comprehensive method for studying protein-ligand interactions. As a result, we employed fluorescence spectroscopy to investigate the interaction between BSA and eupatorin. Generally, BSA fluorescence absorption originates from Trp, Tyr, and Phe residues, whereas its intrinsic fluorescence can be mainly attributed to the Trp residue alone [22].

**Figure 2:**
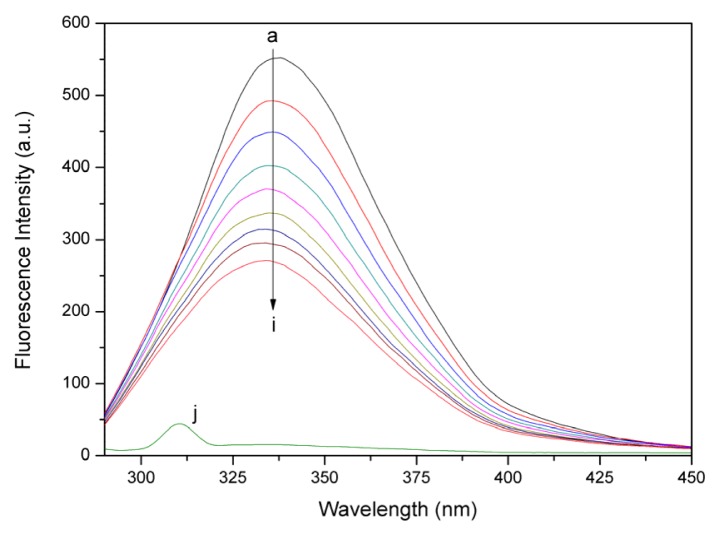
Fluorescence spectra of BSA with various amounts of eupatorin (pH 7.4, 298 K). (**a**) 3.0 × 10^−7^ M BSA; (**b**–**i**) 3.0 × 10^−7^ M BSA in the presence of 2.0, 4.0, 6.0, 8.0, 10.0, 12.0, 14.0, and 16.0 × 10^−7^ M eupatorin; (**j**) 15.0 × 10^−7^ M eupatorin; λ_ex_ = 280 nm, λ_em_ = 290–500 nm.

The fluorescence spectra of BSA with different concentrations of eupatorin were determined and have been shown in Figure 2. BSA fluorescence intensity decreased remarkably as eupatorin concentrations increased and no emission spectral shifting was observed upon BSA-eupatorin complexation. This indicated that eupatorin could interact with BSA, and that the fluorescence chromophores of BSA were not exposed to an obvious polarity change with eupatorin titration.

### 2.3. Quenching Mechanism Analysis

Fluorescence quenching could often be distinguished by its specific temperature dependency. Higher temperature could result in a more rapid diffusion and larger amount of collision quenching, and would typically contribute to the dissociation of a weaker bound complex and to a smaller degree of static quenching [22]. Therefore, increasing the temperature, the quenching constant increases for dynamic quenching and decreases for static quenching. The maximum scatter collision quenching constant reported for various kinds of quencher to a biopolymer is 2.0 × 10^10^ mol^−1^s^−1^[23]. The quenching mechanism induced by eupatorin was further confirmed by the temperature dependency of fluorescence quenching, and the data were analyzed using the Stern–Volmer equation [24]:

(1) $$F_{0}/F=1+K_{SV}\;[Q]=1+k_{q}\tau_{0}[Q]$$

where *F*_0_ and *F* are the steady-state fluorescence intensities in the absence and presence of quencher, respectively; [*Q*] is the concentration of quencher; *k_q_* is the quenching rate constant of biomolecule; τ_0_ is the average lifetime of the protein without the quencher, which is of the order of 10^−8^ s [25]; and *K_sv_* is the Stern–Volmer dynamic quenching rate constant.

**Figure 3:**
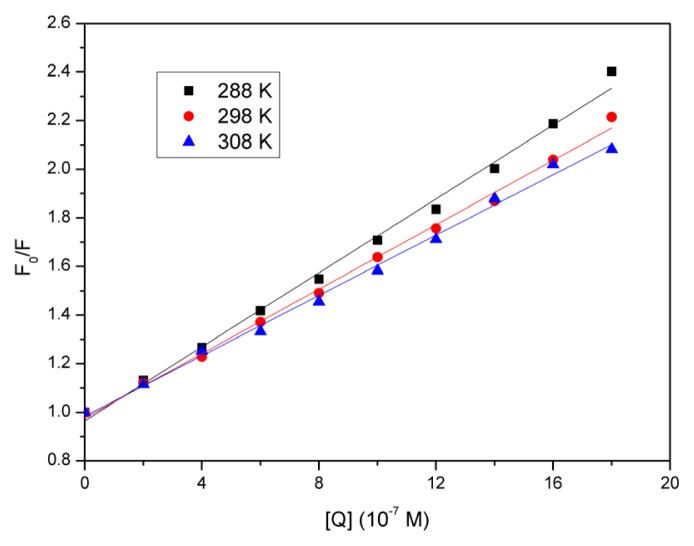
Stern-Volmer plots for quenching of BSA fluorescence by eupatorin at different temperatures (288 K, 298 K and 308 K). *C*_BSA_ = 3.0 × 10^−7^ M; pH 7.4; λ_ex_ = 280 nm, λ_em_ = 290–500 nm.

The fluorescence data were analyzed at three different temperatures, and the Stern–Volmer plots at different temperatures have been shown in Figure 3. A good linear relationship between *F*_0_/*F* and [*Q*] was observed, and the slopes decreased as temperature increased. The *K_sv_* and *k_q_* values derived from Equation (1) at the three temperatures have been presented in Table 1. It could be observed that *K_sv_* and *k_q_* values were inversely correlated with temperature. Further, *k_q_* was much larger than the maximum scatter collision quenching constant, 2.0 × 10^10^ mol^−1^s^−1^, mentioned above. It could thus be assumed that the quenching mechanism was due to complex formation between eupatorin and BSA, rather than dynamic collision. In other words, the BSA fluorescence quenching that was caused by eupatorin resulted from specific complex formation, so dynamic collision effects, if any, should have been negligible. Additionally, the quenching process was further analyzed using the following modified Stern–Volmer equation [26]:

(2) $$\frac{F_{0}}{\Delta F}=\frac{F_{0}}{F_{0}-F}=\frac{1}{f_{a}K_{a}}\frac{1}{\left[Q\right]}+\frac{1}{f_{a}}$$

where, in the present case, *F*_0_ and *F* are the fluorescence intensity in the absence and presence of the quencher, respectively; Δ*F* is the difference in fluorescence intensity of *F*_0_ and *F; K_a_* is the effective quenching constant for the accessible fluorophores, which is analogous to the association binding constants for the quencher-acceptor system; [*Q*] is the concentration of the quencher; and *f_a_* is the fraction of accessible fluorescence.

**Figure 4:**
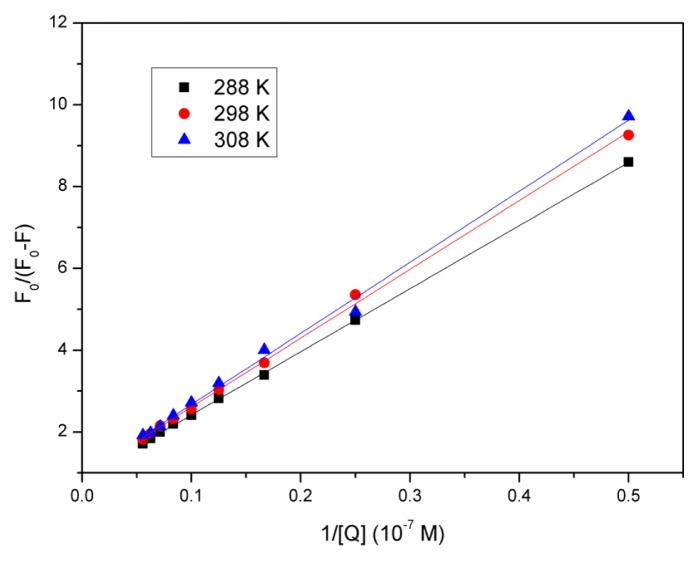
The modified Stern-Volmer plots of BSA at different temperatures in presence of eupatorin; λ_ex_ = 280 nm, λ_em_ = 290–500 nm.

As shown in Figure 4, the curves of *F*_0_/*F versus* [*Q*]^−1^ were linear under a certain quencher concentration. The corresponding parameters have been presented in Table 2. As temperature increased, the decreasing trend of *K_a_* was in accordance with *K_sv_*’s dependency on temperature mentioned above, which coincided with a static quenching mechanism. The results indicated that the binding of eupatorin to BSA was reduced as temperature increased.

### 2.4. Evaluation of the Binding Constant and the Number of Binding Sites

**Figure 5:**
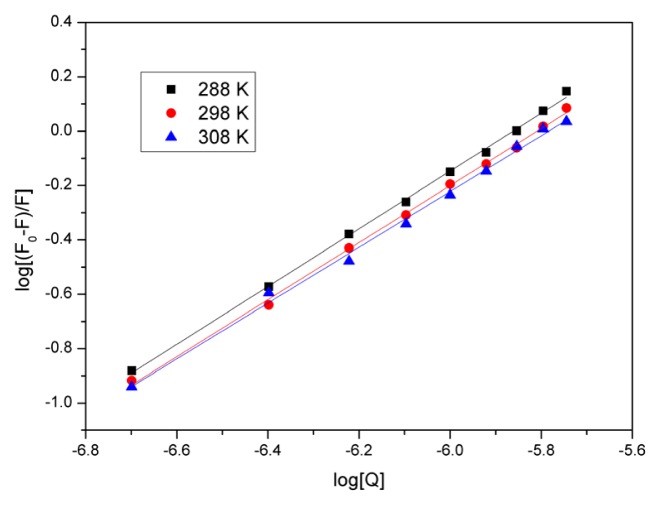
Plots of log[(*F*_0_ − *F*)/*F*] *versus* log[*Q*] for eupatorin quenching effect on BSA fluorescence at different temperatures. *C*_BSA_ = 3.0 × 10^−7^ M; pH 7.4; λ_ex_ = 280 nm, λ_em_ = 290–500 nm.

In static quenching, if similar and independent binding sites in the biomolecule are assumed, the binding constant and number of binding sites may be calculated as follows [27]:

(3) $$\text{log}[(F_{0}-F)/F]=\text{log }K_{b}+n\;\text{log}[Q]$$

where, in the present case, *K_b_* is the binding constant, and *n* is the number of the binding sites per BSA, which can be determined by the ordinate and slope of the double logarithm regression curve (Figure 5) of log[(*F*_0_ − *F*)/*F*] *versus* log[*Q*], based on Equation (3), respectively. The values of *K_b_* and *n* were evaluated and have been presented in Table 3.

The number of binding sites (*n*) was approximately 1, indicating that there was one BSA binding site for eupatorin. The value also showed that *K_b_* and *n* decreased as temperature increased. This was in accordance with the aforementioned trend of *K_sv_* and *K_a_*, and might imply that an unstable complex was formed during the binding. It is possible that the complex would be partly dissociated with increasing temperature.

### 2.5. Thermodynamic Parameters and Nature of the Binding Forces

**Figure 6:**
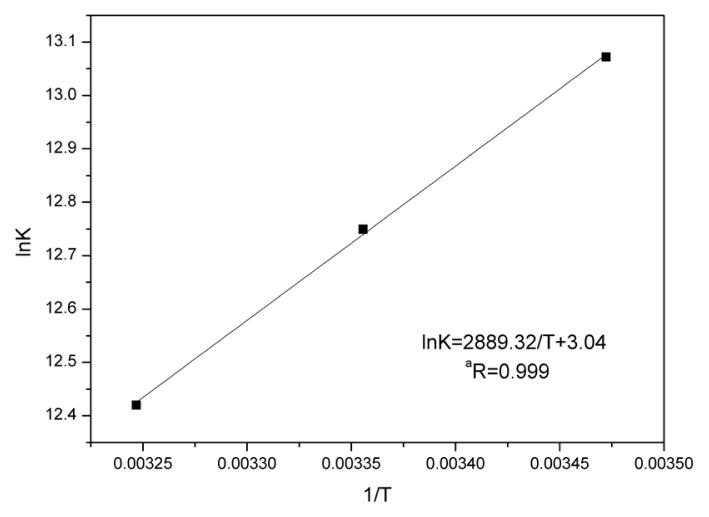
Van’t Hoff plot for the interaction between BSA and eupatorin (pH 7.4). *C*_BSA_ = 3.0 × 10^−7^ M, pH 7.4; λ_ex_ = 280 nm, λ_em_ = 290–500 nm.

Four main types of non-covalent interactions occur in ligand-protein binding: hydrogen bonds, Van der Waals interactions, electrostatic interactions, and hydrophobic forces [28]. Thermodynamic parameters of the binding reaction provide the majority of the evidence to confirm the binding force. Therefore, the temperature-dependent thermodynamic parameters were analyzed in an effort to characterize the acting forces between eupatorin and BSA. If the Δ*H*^0^ (enthalpy change) slightly changes in the temperature range analyzed, then it can be considered constant. Subsequently, its value, and that of Δ*S*^0^ (entropy change), can be calculated using the Van’t Hoff Equation (4). Therefore, the value of Δ*G*^0^ (free energy change) at different temperatures can be derived from Equation (5):

(4) $$\text{ln }K=-\Delta H^{0}/RT+\Delta S^{0}/R$$

(5) $$\Delta G^{0}=\Delta H^{0}-T\Delta S^{0}$$

where, *K* is the binding constant at a corresponding temperature; *R* is the gas constant; and *T* is the absolute temperature. The Δ*H*^0^ and Δ*S*^0^ were obtained from the slope and intercept of the linear Van’t Hoff plot (shown in Figure 6) of ln *K versus* 1/*T*, based on Equation (4).

The values of Δ*H*^0^, Δ*S*^0^, and Δ*G*^0^ have been listed in Table 4. It could be seen that Δ*H*^0^ = −24.02 kJ/mol, and Δ*S*^0^ = 25.31 J/mol·K, which indicated that the binding process was an exothermic reaction accompanied by a negative Δ*H*^0^ value. From the perspective of water structure, a positive Δ*S*^0^ value for a drug–protein interaction is typically regarded as evidence of a hydrophobic interaction [29], as the water molecules that had been arranged in an orderly fashion around the drug and proteins have acquired a more random configuration. We could not attribute the negative Δ*H*^0^ value observed here mainly to electrostatic interactions, because Δ*H*^0^ was close to zero for electrostatic interactions [30,31]. Thus, it was not possible to account for the thermodynamic parameters using a single intermolecular force model. Collectively, hydrophobic and electrostatic interactions most likely play major roles in BSA-eupatorin binding, however, hydrogen bonding could not be excluded as a possible acting force [28]. Meanwhile, the negative value of Δ*G*^0^ indicated the spontaneity of the binding between eupatorin and BSA.

### 2.6. Energy Transfer from BSA to Eupatorin

Fluorescence resonance energy transfer (FRET) is a distance–dependent interaction between the different electronic excited states of molecules. In this interaction, excitation energy is transferred from one molecule (donor) to another (acceptor) through direct electrodynamic interaction, without emission of a photon from the former molecular system [32]. Energy transfer may occur under the following conditions: when the donor can produce fluorescent light; when there is an overlap between the fluorescence emission spectrum of the donor and the absorbance spectrum of the acceptor; and when the distance between the donor and the acceptor is less than 8 nm [28].

**Figure 7:**
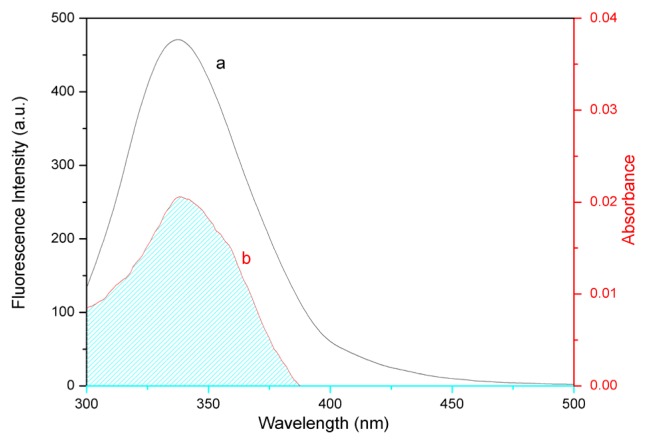
The overlap of the fluorescence emission spectrum of BSA (**a**) with the absorption spectrum of eupatorin; (**b**) *C*_BSA_ = *C*_eupatorin_ = 3.0 × 10^−7^ M, *T* = 298 K, and pH 7.4; λ_ex_ = 280 nm, λ_em_ = 290–500 nm.

The efficiency of energy transfer between eupatorin and the BSA Trp213 residue could be used to evaluate the distance between the two using FRET. The overlap of the absorbance spectrum of eupatorin with the fluorescence emission spectrum of BSA has been shown in Figure 7.

According to Förster’s non-radiative resonance energy transfer theory [33,34], energy transfer efficiency *E* is related not only to the distance (*r*) between the bound drug (acceptor) and the protein residue (donor), but also to the critical energy transfer distance (*R*_0_). By Förster’s theory, the efficiency of energy transfer (*E*) can be calculated according to the following equation:

(6) $$E=1-F/F_{0}=R_{0}^{6}/(R_{0}^{6}+r^{6})$$

where *r* is the distance between acceptor (eupatorin) and donor (BSA), and *R_0_* is the critical distance when the transfer efficiency is 50%. The value of *R_0_* is calculated using the following equation:

(7) $$R_{0}^{6}=8.79\times 10^{-25}K^{2}n^{-4}\phi J$$

where *K*^2^ is the spatial orientation factor of the dipole; *n* is the refractive index of the medium; ϕ is the fluorescence quantum yield of donor; and *J* is the spectral overlap between the emission spectrum of donor and the absorption spectrum of acceptor (Figure 7), given by:

(8) $$J=\frac{\int_{0}^{\infty }F(\lambda )ɛ(\lambda )\lambda^{4}\text{d}\lambda }{\int_{0}^{\infty }F(\lambda )\text{d}\lambda }$$

where *F*_(λ)_ is the fluorescence intensity of the donor at wavelength λ, and ɛ(λ) is the molar absorption coefficient of the acceptor at wavelength λ. In the present case, *K^2^* = 2/3, *n* = 1.36, and ϕ = 0.15 [35]. Hence, from Equations (6)–(8), we could calculate the following parameters: *J* = 1.79 × 10^−14^ cm^3^ L mol^−1^, *R*_0_ = 1.56 nm, and *r* = 1.64 nm. In conclusion, the distance (*r*) between BSA Trp213 and bound eupatorin was much less than 8 nm, and accorded with the relationship 0.5*R*_0_ < *r* <1.5*R*_0_. This implied that the non-radiative energy transfer from BSA to eupatorin occurred with high possibility, which was in accordance with the occurrence of a static quenching mechanism. This result indicated that the binding obeyed the conditions of Förster’s energy transfer theory, and verified that eupatorin is located in domain II of BSA, where Trp213 was located [14].

### 2.7. Analysis of BSA Conformational Changes

#### 2.7.1. Synchronous Fluorescence Spectroscopic Studies

Synchronous fluorescence spectroscopy is a method that is widely used to explore the microenvironment of amino acid residues via measurement of the emission wavelength shift [36]. The process is beneficial because of its characteristic sensitivity, spectral simplification, spectral bandwidth reduction, and avoidance of perturbing effects [37]. Vekshin found this method to be of great use for studying the environment of amino acid residues via measurement of the possible shift in wavelength emission maximum, lmax, and the shift in position of emission maximum corresponding to the changes of the polarity around the chromophore molecule [38].

**Figure 8:**
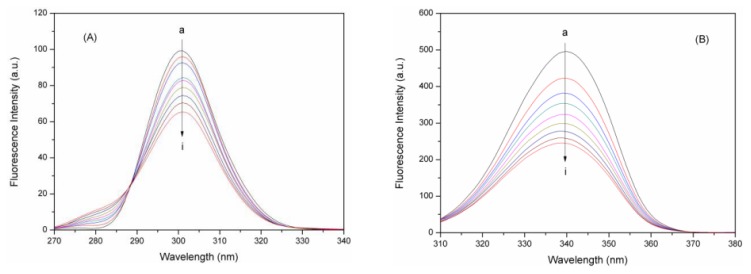
Synchronous fluorescence spectra of BSA with various amounts of eupatorin. (pH 7.4, *T* = 298 K): (**A**) Δλ = 15 nm (λ_ex_ = 240nm, λ_em_ = 255–400 nm); (**B**) Δλ = 60 nm (λ_ex_ = 240 nm, λ_em_ = 300–400 nm). *C*_BSA_ = 3.0 × 10^−7^ M; *C*_eupatorin_ = 0.0, 2.0, 4.0, 6.0, 8.0, 10.0, 12.0, 14.0, and 16.0 × 10^−7^ M, respectively.

As is well known, the synchronous fluorescence spectra of BSA provides the characteristic information for the Try residues and Trp residues [39] when the wavelength interval Δλ (Δλ = λ_em_ − λ_ex_) is fixed at 15 and 60 nm, respectively. Figure 8A,B have shown the synchronous fluorescence spectra of Tyr and Trp residues in BSA with various amounts of eupatorin, respectively. The BSA fluorescence intensity regularly decreased with the addition of eupatorin. This further demonstrated the occurrence of fluorescence quenching in the binding. Moreover, there was no significant shift of the maximum emission wavelength at Δλ = 15 nm (Figure 8A) or 60 nm (Figure 8B), implying that the interaction of eupatorin and BSA could not affect the microenvironment around the Tyr or Trp residues.

#### 2.7.2. Changes in the BSA Secondary Structure Induced by Eupatorin Binding

**Figure 9:**
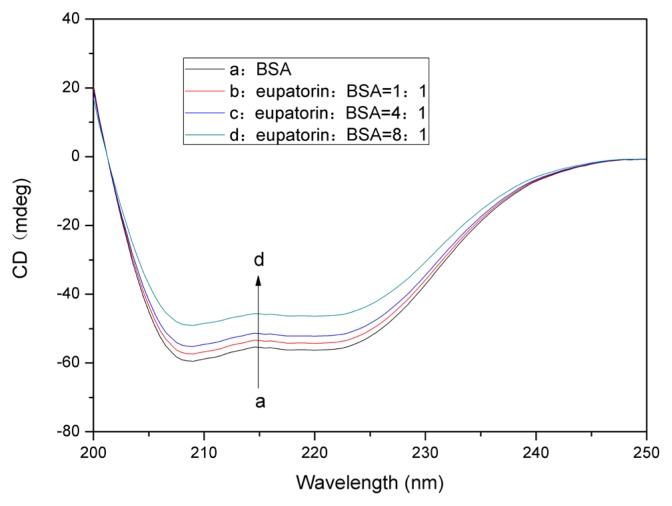
CD spectra of the eupatorin–BSA system (pH 7.4, *T* = 310 K). *C*_BSA_ = 5.0 × 10^−7^ M; eupatorin to BSA ratios: (**a**) 0:1; (**b**) 1:1; (**c**) 4:1; and (**d**) 8:1.

Circular dichroism (CD) is a sensitive and powerful technique that is used to explore the conformational changes in proteins induced by a ligand. The CD spectra of BSA are typically characterized by two negative bands at 208 nm and 222 nm, which are rationalized by the *n*→π* transition [40], the so-called negative Cotton effect [41,42], in the peptide bond of α-helix. To better understand the eupatorin–BSA binding mechanism and the secondary structure changes of BSA, further CD measurements were carried out on the BSA and eupatorin-BSA complex. The CD spectra of BSA at pH 7.4 in the absence and presence of eupatorin have been presented in Figure 9. Two negative bands could be observed at 208 and 222 nm. The CD results were expressed in terms of mean residue ellipticity (MRE) in deg cm^2^ dmol^−1^, according to the following equation [43,44]:

(9) MRE=Observed CD(mdeg)/(10Cpnl)

where, *n* is the number of amino acid residues (583); *l* is the path length of the cell (1 cm); and Cp is the molar concentration. The helix content was calculated from the MRE values at 208 nm, according to the following equation, which was described in a prior study [44]:

(10) $$\alpha -\text{Helix}(\%)=[(-{\text{MRE}}_{208}-4000)\times 100]/(33000-4000)$$

where, MRE_208_ is the observed MRE value at 208 nm; 4000 is the MRE value for the random coil conformation at 208 nm; and 33,000 is the MRE value of a pure α-helix at 208 nm. The α-helix content in the secondary structure of BSA could be calculated according to the equation above. A significant reduction in negative ellipticity without any significant shift of the peaks was observed in presence of eupatorin, demonstrating the decrease of α-helix content in the protein (Figure 9). It was evident that the interaction of eupatorin and BSA caused the conformational changes of BSA, thus leading to a loss of α-helix stability. The calculated results showed a reduction of α-helix content from 56.5% in free BSA to 54.1%, 51.5%, and 44.2% at eupatorin/BSA molar ratios of 1:1, 4:1, and 8:1, respectively.

### 2.8. Docking Analysis

**Figure 10:**
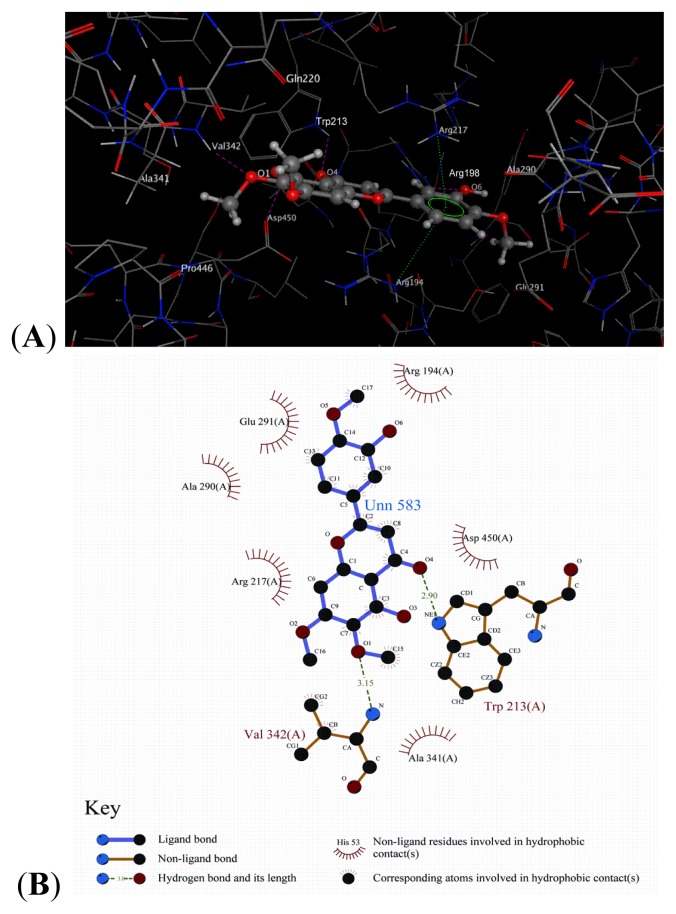
(**A**) Three dimensional view of eupatorin binding site. The enlargement of ligand in the binding site; the ligand is depicted by balls and sticks, hydrogen bonds are depicted by pink dashed lines, and Van der Waals interactions are depicted by green dashed lines; (**B**) Two-dimensional schematic representation of hydrogen bond interactions.

Molecular docking was employed to understand the interaction between BSA and eupatorin and to ultimately elucidate the interaction mechanism. Results obtained by AutoDock tools presented 10 different conformations of eupatorin. We selected the best conformation for further analysis, owing to its higher binding affinity (−20.46 kJ/mol) and lowest RMSD value (0 Å). The analysis showed that Val342, Asp450, Trp213, Arg198, Arg194, Arg217, Gln220, Ala341, Pro446, Ala290, and Glu291 were the most vital residues present at the active site. The possible interacting model and the main residues involved in the interaction have been depicted in Figure 10A,B. The ligand core contacting with the protein was anchored in the binding site by H-bonds. The three oxygen atoms at the O1, O4, and O6 positions form three H-bonds with the NH of Val342 (−O…NH, 2.08 Å, 158.5°), of Trp213 (−O…NH, 2.12 Å, 125.2°), and of Arg198 (−O…NH, 2.29 Å, 131.2°), respectively. Meanwhile, the hydroxyl oxygen atoms at the O3 position of eupatorin act as hydrogen donor to form H–bond with the carbonyl group of Asp450 (−O…OH, 2.04 Å, 105.7°). We found that the arene of the eupatorin formed an arene-cation through contact with the −NH_2_ of the Arg194 and Arg217. The Gln220 and Glu291 residues interacted with eupatorin through polar contact. However, a series of hydrophobic residues, Ala341, Pro446, and Ala290, around the peripheral region of the molecule interacted with eupatorin through hydrophobic interactions.

The human serum albumin (HSA) is homologous to BSA. One study of HSA inhibitor thyroxine indicated that its binding position was approximately the same as that of eupatorin [45]. From the docking analysis, we could presume that the hydrophobic interactions and the polar contacts collectively constituted the primary force for the binding of the molecule, which further verified the results of Section 2.5. Collectively, these results demonstrated the acceptability of this model.

## 3. Experimental Section

### 3.1. Materials

BSA fraction V and eupatorin were purchased from the Sigma Chemical Company (St. Louis, MO, USA) and used without further purification. All other reagents were of analytical grade. Double-distilled water was used throughout the experiments. BSA solutions were prepared in 0.02 M phosphate buffered saline (PBS) at pH 7.4, and ion strengths were kept at 0.1 M. A 3.0 × 10^−3^ M ethanol solution of eupatorin was used for all binding experiments, and percentage of ethanol dissolved with eupatorin in BSA solution was under 3% in order to ensure that ethanol has no effect on structure of BSA [46–48]. All solutions were stored in a refrigerator at 4 °C in the dark.

### 3.2. Apparatus

UV–vis absorption spectroscopy was conducted on a UV-2550 spectrophotometer (Shimadzu, Kyoto, Japan) equipped with 1.0 cm quartz cells.

Fluorescence measurements were performed on an RF-5301PC spectrofluorophotometer (Shimadzu, Kyoto, Japan) equipped with 1.0 cm quartz cells.

CD measurements were made on a J-810 spectropolarimeter (JASCO, Tokyo, Japan) equipped with 1.0 cm quartz cells.

The pH value of the buffer solution was measured with a PB-10 exact digital pH meter (Sartorius, Göttingen, Germany).

Data were analyzed using Origin 8.0 software (OriginLab Corporation, Northampton, MA, USA).

### 3.3. Procedures

#### 3.3.1. Measurements of UV–vis Absorption Spectra

BSA UV–vis measurements in the presence and absence of eupatorin were conducted in the range of 200–500 nm. The BSA concentration was fixed at 1.0 × 10^−5^ M, while the eupatorin concentration was 1.0 × 10^−5^ M. Following the addition of BSA and eupatorin, the solution was equilibrated for 5 min, and absorbance values were recorded thereafter.

#### 3.3.2. Measurements of Fluorescence Spectra

In this assay, a 3.0 mL solution with a fixed concentration of 3.0 × 10^−7^ M BSA was accurately added into the quartz cell with a 1 cm path length, and was manually titrated by successive additions of 2.0 × 10^−4^ M eupatorin at 5 min time intervals. The fluorescence emission spectra were then measured at three different temperatures (288, 298, and 308 K). Spectra were recorded in the wavelength range of 290–500 nm setting the excitation at 280 nm, and excitation and emission bandwidths at 5 nm. Each spectrum was the mean of at least three scans. Fluorescence intensities were corrected for inner filter effect, according to the following equation [49,50]:

(11) $$F_{cor}=F_{obs}\;\text{exp}[(A_{ex}+A_{em})/2]$$

where, *F_cor_* and *F_obs_* are the corrected and observed fluorescence intensity, respectively; and *A_ex_* and *A_em_* are the absorbance at excitation and emission wavelengths, respectively.

There exists absorbance in eupatorin, which could be seen in Figure 1, the results of UV–vis. To avoid the effects of the phenomena on fluorescence results, the following equation was applied [51,52]:

(12) $$F=F_{u}e^{-2.303ɛ{IL}_{0}}$$

where, *F* is the corrected fluorescence, *F_u_* is the experimental fluorescence, ɛ is the molar extinction coefficient of the ligand, *I* is the pathlength and *L*_0_ is the ligand concentration.

#### 3.3.3. Measurements of Synchronous Fluorescence Spectra

Samples used here were the same as those described in section 3.3.2 (the measurements of fluorescence spectra). Synchronous fluorescence spectra were obtained by simultaneously scanning the excitation and emission spectra. The wavelength interval between the emission and excitation wavelength was individually fixed at 15 and 60 nm, at which the spectrum only showed the spectroscopic behavior of Tyr and Trp residues of BSA, respectively. The excitation wavelength is 240 nm for both Tyr and Trp residues of BSA, and the emission wavelength is 255–400 nm and 300–400 nm for Tyr and Trp residues of BSA, respectively. Each spectrum was the mean of at least three scans, and the data were corrected for the signal of the buffer solution. The fluorescence intensities were corrected using Equations (11) and (12).

#### 3.3.4. Measurements of CD Spectra

BSA CD spectra in the range of 190–250 nm were recorded on a JASCO J-810 automatic recording spectropolarimeter (Tokyo, Japan), controlled by the Jasco software, with a 1.0 cm quartz cell in nitrogen atmosphere. BSA CD measurements in the presence and absence of eupatorin were performed with a scan rate of 50 nm/min. A stock solution of 5.0 × 10^−7^ M BSA was prepared in PBS. The BSA concentration was held constant (5.0 × 10^−7^ M), while the eupatorin concentration was varied (0, 0.5, 2.0, and 4.0 × 10^−6^ M in ethanolic solution). The buffer solution was chosen as the blank, and was manually subtracted from each spectrum during scanning. Each sample was scanned three times and averaged for a CD spectrum.

### 3.4. Docking study

For the molecular docking study, the crystal structure of BSA (PDB entry code 3V03) was downloaded from the RSCB Protein Data Bank (http://www.rscb.org), and ligand structures were obtained from the PubChem database (http://pubchem.ncbi.nlm.nih.gov). The AutoDock program [53,54] was used to locate the appropriate binding orientations and ligand conformations in the BSA binding pocket [14]. AutoDocktools (ADT) version 1.5.4 (http://mgltools.scripps.edu) was employed to prepare both the ligand and the receptors for docking. The protein was checked for polar hydrogen, and partial atomic Kollman charges were assigned. Torsion bonds of the inhibitor were selected and defined. Then, the three-dimensional grid box was created by the AutoGrid program (part of the AutoDock software package). The grid box (38 × 44 × 54) was generated according to the Trp213 residue [11,12]. It had a distance of 15 Å to the ligand, which essentially coincided with the result described in Section 2.6. Further, the box covered the protein pocket where the binding cavity was located. The spacing value was adjusted to 0.375 Å. Finally, AutoDock was used to calculate the binding free energy of a given inhibitor conformation in the macromolecular structure. The standard docking protocol for rigid protein and flexible ligand docking consisted of the following: 50 independent runs using an initial population of 50 randomly placed individuals, with 2.5 × 10^6^ energy evaluations; a maximum number of 27,000 iterations; a mutation rate of 0.02; a crossover rate of 0.80; and an elitism value of 1. Random starting positions, random orientations, and random torsions were used for the ligands. After docking, the 50 solutions were clustered into groups with RMS deviations under 1.0 Å. Clusters were ranked according to the lowest energy representative of each cluster. The optimal conformation was selected based on the highest binding affinity value (it can also be selected on the basis of the root mean square value from a reference structure given in the log file generated by AutoDock). The crystallographic conformation of compound thyroxine in HSA (PDB code 1hk1) was utilized as a reference to evaluate the accuracy of the predicted model [45]. Visual observance in PyMOL [55] was employed to help find and select a conformation that could properly bind to the protein pocket.

## 4. Conclusions

In this study, the interaction between eupatorin and BSA was investigated *in vitro* using spectroscopic and molecular modeling methods under physiological conditions. The results showed that hydrophobic forces, electrostatic interactions, and hydrogen bonds played vital roles in the eupatorin-BSA binding interaction, which was spontaneous. During the interaction, eupatorin could insert into the hydrophobic pocket, where the non-radioactive energy transfer from BSA to eupatorin occurred with high possibility. The binding distance (*r*) between eupatorin and the BSA Trp213 residue was calculated as ~1.64 nm (298 K). There was no significant change in the microenvironment of Tyr and Trp residues, and the BSA secondary structure was changed after being bound with eupatorin, resulting in the decline of α-helix content. The results of the spectral investigations were further verified by the molecular docking method. The docking method provided a means to estimate the participation and interactions of specific chemical groups in the process of complex stabilization at the molecular level. In summary, this study may provide valuable insight into the binding mechanism of eupatorin and BSA, and could provide a better understanding of the drug’s effect on protein function during the course of its transportation and distribution in the blood. Ultimately, this information could contribute to the pharmaceutical development and application of eupatorin.

## Figures and Tables

**Table 1:** Stern-Volmer quenching constant *K_sv_* and bimolecular quenching rate constant *K_q_* of the BSA-eupatorin system at different temperatures; λ_ex_ = 280 nm, λ_em_ = 290–500 nm.

pH	*T* (K)	*K_sv_* (10^5^ M^−1^)	*K_q_* (10^13^ M^−1^s^−1^)	*R*^a^
7.4	288	7.60	7.60	0.994
	298	6.63	6.63	0.996
	308	6.21	6.21	0.995

a *R* is the linear correlation coefficient.

**Table 2:** Modified Stern-Volmer association constant *K_a_* at pH7.4; λ_ex_ = 280 nm, λ_em_ = 290–500 nm.

pH	*T* (K)	*K_q_* (10^5^ M^−1^)	*R*^a^
7.4	288	5.68	0.999
	298	5.54	0.998
	308	5.39	0.996

a *R* is the linear correlation coefficient.

**Table 3:** Binding constant *K_b_* and the number of binding sites *n* at different temperatures; λ_ex_ = 280 nm, λ_em_ = 290–500 nm.

pH	*T* (K)	*K_b_* (10^6^ M^−1^)	*n*	*R*^a^
7.4	288	1.679	1.062	0.998
	298	1.230	1.048	0.998
	308	0.857	1.026	0.995

a *R* is the linear correlation coefficient.

**Table 4:** Thermodynamic parameters for eupatorin-BSA interaction at pH 7.4; λ_ex_ = 280 nm, λ_em_ = 290–500 nm.

pH	*T* (K)	Δ*G*^0^ (kJ/mol)	Δ*H*^0^ (kJ/mol)	Δ*S*^0^ (J/mol·K)	*R*^a^
7.4	288	−31.31			
	298	−31.56	−24.02	25.31	0.999
	308	−31.82			

a *R* is the linear correlation coefficient.
